# A neutron track etch detector for electron linear accelerators in radiotherapy

**DOI:** 10.2478/v10019-010-0003-2

**Published:** 2010-03-18

**Authors:** Branko Vukovic, Dario Faj, Marina Poje, Maja Varga, Vanja Radolic, Igor Miklavcic, Ana Ivkovic, Josip Planinic

**Affiliations:** 1 Department of Physics, University of Osijek; Osijek, Croatia; 2 Clinical Hospital Osijek, Osijek, Croatia

**Keywords:** electron linear accelerator, photoneutron, track etch detector, neutron dose equivalent

## Abstract

**Background:**

Electron linear accelerators in medical radiotherapy have replaced cobalt and caesium sources of radiation. However, medical accelerators with photon energies over 10 MeV generate undesired fast neutron contamination in a therapeutic X-ray photon beam. Photons with energies above 10 MeV can interact with the atomic nucleus of a high-Z material, of which the target and the head of an accelerator consist, and lead to the neutron ejection.

**Results and conclusions.:**

Our neutron dosimeter, composed of the LR-115 track etch detector and boron foil BN-1 converter, was calibrated on thermal neutrons generated in the nuclear reactor of the Josef Stefan Institute (Slovenia), and applied to dosimetry of undesirable neutrons in photon radiotherapy by the linear accelerator 15 MV Siemens Mevatron. Having considered a high dependence of a cross-section between neutron and boron on neutron energy, and broad neutron spectrum in a photon beam, as well as outside the entrance door to maze of the Mevatron, we developed a method for determining the effective neutron detector response. A neutron dose rate in the photon beam was measured to be 1.96 Sv/h. Outside the Mevatron room the neutron dose rate was 0.62 μSv/h. PACS: 87.52. Ga; 87.53.St; 29.40.Wk.

## Introduction

Nowadays, cobalt and caesium teletherapy machines in medical radiotherapy are being replaced by linear accelerators.^1^ The great advantage of this new equipment is that it has no attached radioactive source, which makes them safer from the radiological point of view. However, medical accelerators with photon energies over 10 MeV generate undesired fast neutron contamination in a therapeutic beam. Photons with energies above 10 MeV can interact with the atomic nucleus of a high-Z material, of which the target and the head of the accelerator consist, and lead to the neutron ejection. Consequently, this can increase the patient dose and pose a problem in room shielding dosimetry, which is essential for the quality assurance in radiotherapy.^2^,^3^

Neutrons are principally produced through giant dipole resonance in a nuclear reaction (γ, n) between photons and target nuclei.^4^ The giant resonance process produces two groups of neutrons; the first and the largest group has a spectrum, which can be described by a Maxwellian distribution, with the most probable energy, typically between 0.4 and 1 MeV; the second group of neutrons is produced by direct emission and is somewhat higher in energy (up to 10–20% of the total neutrons in general).^5^ The mean energy of the neutron spectrum generated by the (γ,n) reaction is around a few MeV, but, at the patient plane, neutrons have a more complex distribution and a less mean energy. As beam energies increase (>10 MeV), an undesirable photoneutron dose also increases. Otherwise one can expect, that inside the area treated by linear accelerator, the neutron dose in a tissue will not exceed 1% of the photon dose.^6^

## Material and methods

The neutron dosimeter consisted of the LR-115 track detector and boron foil BN-1 (Kodak-Pathe, France) or ^10^B converter for reaction (n, α); it was calibrated on neutrons generated in the nuclear reactor of the J. Stefan Institute (Slovenia).^7^ Neutron irradiation was carried out in the thermal column of the TRIGA Mark II reactor where the neutron flux was Φ = 3.33×10^5^ cm^−2^ s^−1^; for irradiation time, t = 240 s, we got the fluence, f = Φt = 7.99×10^7^ cm^−2^.

The LR-115 detectors, 2×3 cm^2^, were etched in a 10% NaOH aqueous solution at 60 °C for 120 min and afterwards counted visually using a microscope of (10×16) magnification.

The irradiated neutron track etch detector had a net track density D_t_ = (63394 – B) ≅ 63370 cm^−2^, where the background, B, was 24 cm^−2^; the standard deviation of the D_t_ was s_Dt_ = 570. The response, r_t_, of the neutron dosimeter for thermal neutrons was:

[1] $${\text{r}}_{\text{t}}={\text{D}}_{\text{t}}/\text{f}=(7.930\pm 0.071)\times 10^{-4},$$

where the error was given as a standard deviation, s_rt_ = s_Dt_/f = 7.1×10^−6^.

The electron linear accelerator Siemens Mevatron 15 MV has been used as an X-ray radiotherapy source in the Clinical Hospital Osijek. The same accelerator was used to perform experiments for determining a dose equivalent of undesirable photoneutrons by using the neutron track etch detector.

## Results and discussion

### Linear accelerator neutrons – track detector in the beam

In order to determine a dose equivalent of photoneutrons produced by linear accelerator, operating in a photon production mode at an acceleration potential of 15 MV, we used the LR-114 track etch detector, which was positioned in the beam at 1 m from the accelerator head.

**FIGURE 1:**
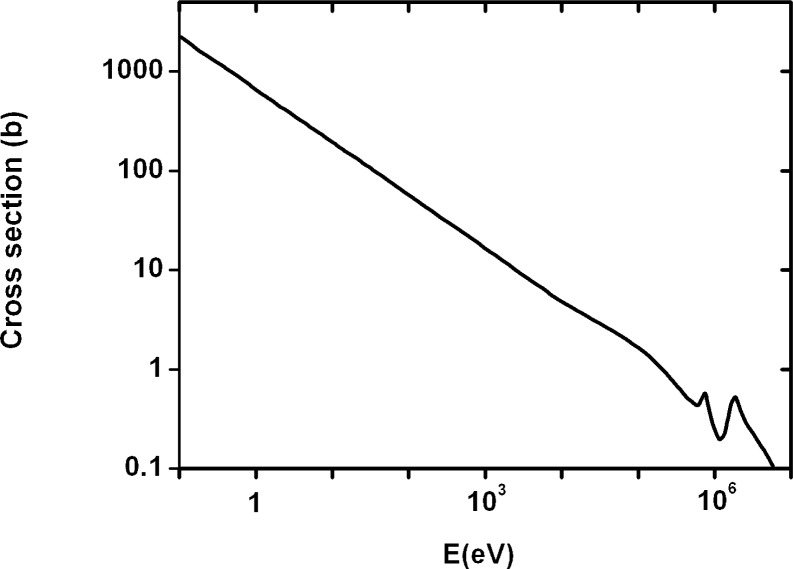
The total cross-sections of neutrons on boron (σ/b) versus neutron energy (E/eV).^8^

**FIGURE 2:**
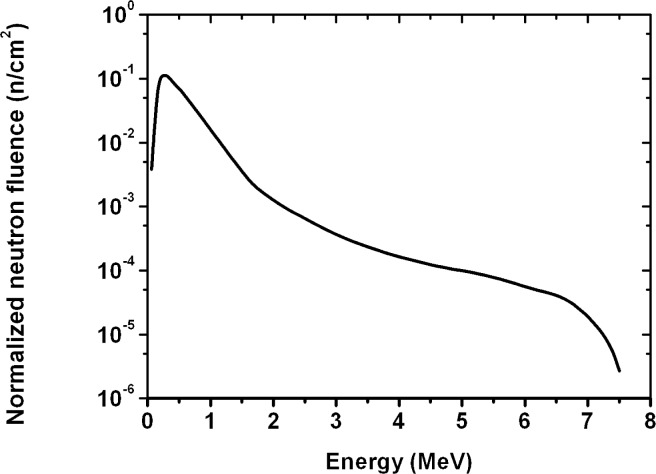
Neutron spectrum produced in 15 MV linear accelerator after crossing the tungsten head; fluence (f/n cm^−2^) versus neutron energy (E/MeV).^2^

For fast neutrons with higher energy, a detector response should be lower; having considered a total cross-section of neutrons on boron, ^10^B (n, α) ^7^Li, depending on neutron energy (σ(E), Figure 1)^8^, as well as a neutron fluence spectrum on energies by the linear accelerator (Figure 2)^2^, we divided neutron energy E_a_ (MeV) in the two areas as follows: the first area, 0 < E_a1_ < 0.5, and the second one, 0.5 ≤ E_a2_ ≤ 7.5.

Afterwards, we estimated the respective mean cross-sections σ_t_ = 1000 b (for thermal neutrons), σ_a1_ = 1.33 b and σ_a2_ = 0.25 b (for energies E_a1_ and E_a2_, respectively) from the curve σ(E) in Figure 1, and we determined the neutron detector responses for the tow energy areas as follows: σ_t_/σ_a1_ = r_t_/r_a1_, σ_t_/σ_a2_ = r_t_/r_a2_, where the r_t_ was the response that had already been calculated by using equation [1]. Thus we got r_a1_ = 1.06 × 10^−6^ and r_a2_ = 2.00 × 10^−7^, with the standard deviations of 9.1×10^−9^ and 1.8×10^−9^, respectively, and we were able to determine an average or effective value of the neutron detector response r_ae_, but as a weighted or pondered mean.^9^

Therefore, we took the surfaces under the neutron fluence spectrum curve (Figure 2; f(E)) for the two energy areas as above and we got the relative surfaces: s_a1_ = 0.58 and s_a2_ = 0.42, those had the meaning of the relative frequencies in the calculation of the pondered mean (for instance, s_1_ is the ratio of the surface under the curve between 0 and 0.5 MeV to the total surface under the curve between 0 and 7.5 MeV). The mean or effective detector response was: r_ae_ = r_a1_s_a1_ + r_a2_ s_a2_ = (6.97 ± 0.07) ×10^−7^.

For a measured detector density D_a_, the respective fluence was (like in equation [1]): f_ae_ = D_a_ /r_ae_. When the neutron fluence is known, a conversion coefficient (*k*) from neutron fluence to dose equivalent, depending on neutron energy, gives a personal dose equivalent (H_a_), as follows:^10^ H_a_= k_ae_ f_ae_.

**FIGURE 3:**
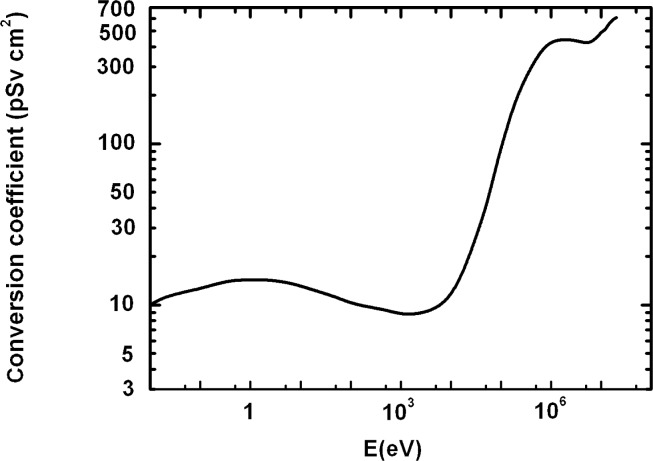
Conversion coefficient (k/pSv cm^2^) from neutron fluence to personal dose equivalent versus neutron energy (E/eV).

Having considered a great dependence of *k* on neutron energy (Figure 3), we calculated the average or effective *k*_ae_ for the two energy areas, like above, and we used the same relative frequencies s_ai_ (i = 1, 2); taking the average values of *k*_a_ for the neutron energies E_a1_ and E_a2_ as k_a1_ = 200 and k_a2_ = 430 pSv cm^2^, respectively, we calculated the effective conversion factor as follows:

$${\text{k}}_{\text{ae}}={\text{k}}_{\text{a}1}{\text{s}}_{\text{a}1}+{\text{k}}_{\text{a2}}{\text{s}}_{\text{a2}}=296.4\;\text{pSv }{\text{cm}}^{2}.$$

Thus, the measured detector net density D_a_ = (383.1 ± 0.04) cm^−2^ corresponded to the following dose equivalent:

[2] $$\begin{array}{l} {\text{H}}_{\text{a}}={\text{k}}_{\text{ae}}{\text{f}}_{\text{ae}}={\text{k}}_{\text{ae}}/{\text{r}}_{\text{ae}}{\text{D}}_{\text{a}}=425.5{\text{D}}_{\text{a}}(\mu \text{Sv}),\\ {\text{H}}_{\text{a}}=(0.163\pm 0.002)\text{Sv} \end{array}$$

The dose rate was calculated as a ratio of the dose equivalent and exposure time, t, or:

- *Ḣ* = H/t, and for t = 5 min, we got the dose rate *Ḣ* = (1.96 ± 0.02) Sv/h.

The measurement errors were determined as variances or standard deviations for track densities in the following way (according to the Poisson distribution):

$${\text{s}}_{\text{D}}{}^{2}={\text{s}}_{\text{Db}}{}^{2}+{\text{s}}_{\text{B}}{}^{2}={\text{D}}_{\text{b}}+\text{B};$$

The dose equivalent variance was calculated as total differential of the function of the form like in equitation [2], which led to the following expression:

$${\text{s}}_{\text{H}}{}^{2}={\left({\text{kD}/\text{r}}^{2}\right)}^{2}{\text{s}}_{\text{r}}{}^{2}+{\left(\text{k}/\text{r}\right)}^{2}{\text{s}}_{\text{D}}{}^{2}.$$

### Linear accelerator neutrons – track detector behind the wall

**FIGURE 4:**
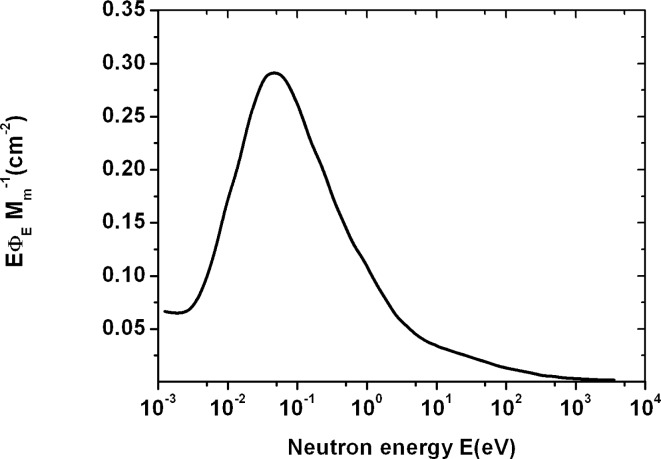
Neutron spectrum measured at position outside the entrance door to the maze of the 15 MV Mevatron.^11^

The neutron track etch detector was positioned outside the entrance door to the accelerator maze. The neutron spectrum in the same position was measured by Schraube *et al*.^11^, hereby presented in Figure 4. We used the given neutron spectrum in the procedure for determining a neutron dose equivalent by the track detector, as above. Otherwise, neutron spectrum can vary depending on the wall construction of a room.

We divided neutron energy, E_b_, in two areas as follows: the first area, 0 < E_b1_ < 100 eV, and the second one, 100 eV ≤ E_b2_ ≤ 3750 eV. Because the E_b1_ was the area of thermal neutrons, we estimated the respective mean cross-sections σ_t_ = σ_b1_ = 120 b and σ_b2_ = 14.75 b (for energies E_b1_ and E_b2_, respectively) from the curve σ(E) in Figure 1, and we determined the neutron detector responses for the low energy areas as follows: σ_b1_/σ_b2_ = r_b1_/r_b2_, where r_b1_ = r_t_ was already assessed response by using equation [1]. Thus we got r_b1_ = 7.94×10^−4^ and r_b2_ = 9.75×10^−5^, and were able to determine the average or effective value of the neutron detector response r_be_ for neutrons in the energy area E_b_.

As in the previous case, we took the surfaces under the neutron fluence spectrum curve (Figure 4; f(E)) for the two energy areas and we got the relative surfaces: s_b1_ = 0.18 and s_b2_ = 0.82, those had the meaning of the relative frequencies in the calculation of the pondered mean. The mean or effective detector response was: r_be_ = r_b1_s_b1_ + r_b2_ s_b2_ = 2.2×10^−4^.

For the measured detector density D_b_, the respective fluence is (like in equation [1]): f_be_ = D_b_ /r_be_. When the neutron fluence is known, a conversion coefficient (*k*) from neutron fluence to dose equivalent, depending on neutron energy, gives a personal dose equivalent (H_a_), as follows: H_b_= k_be_ f_be_.

Having considered a great depending *k* on neutron energy (Figure 3), we calculated the average or effective k_be_ for the two energy regions, like above, and we used the same relative frequencies s_bi_ (i = 1, 2); taking the average values of *k*_b_ for the neutron energies E_b1_ and E_b2_ as k_b1_ = 11.58 and k_b2_ = 9 pSv cm^2^, respectively, we calculated the effective conversion factor as follows:

$${\text{k}}_{\text{be}}={\text{k}}_{\text{b1}}{\text{s}}_{\text{b1}}+{\text{k}}_{\text{b}2}{\text{s}}_{\text{b}2}=9.45\;\text{pSv }{\text{cm}}^{2}.$$

Thus, the measured detector net density D_b_ = 1.2 ± 0.01 cm^−2^ corresponded to the following dose equivalent:

$$\begin{array}{l} {\text{H}}_{\text{b}}={\text{k}}_{\text{be}}{\text{f}}_{\text{be}}={\text{k}}_{\text{be}}/{\text{r}}_{\text{be}}{\text{D}}_{\text{b}}=42.9\times 10^{-3}{\text{D}}_{\text{b}}(\mu \text{Sv}),\\ {\text{H}}_{\text{b}}=(0.050\pm 0.0006)\;\mu \text{Sv}. \end{array}$$

The dose rate was calculated as a ratio of the dose equivalent and exposure time of 5 min, and we got the neutron dose rate outside the entrance door to the maze of the 15 MV Mevatron, *Ḣ_b_* = (0.62 ± 0.007) μSv/h. The linac room 1.7 m walls were constructed of barite concrete, with density of 3200 kg/m^3^.

Although the obtained neutron dose rate outside the accelerator room was 3165 times smaller than the neutron dose rate in the photon beam, the measured dose rate *Ḣ_b_* was not negligible from the aspect of personal dosimetry.

**FIGURE 5:**
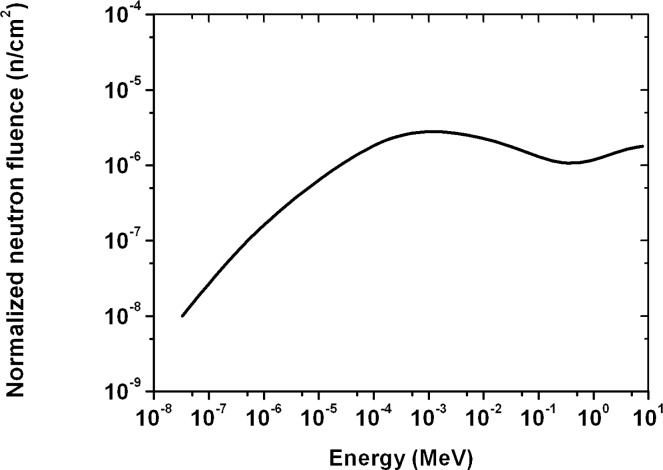
Neutron spectrum attenuation from a 15 MV linear accelerator, after concrete barrier of 1 m.

Some considerations of neutron energy attenuation after crossing the treatment room walls were performed for different concrete barrier thickness and materials. A neutron spectrum attenuation from the 15 MV linear accelerator, after passing a conventional 1 m concrete barrier, with density of 2260 kg/m^3^, was measured by Facure *et al*., hereby presented in Figure 5.^2^

Observing the neutron spectra in Figure 5 and Figure 4, one can notice a broad neutron energy area in Figure 5 (from 0.1 eV to 10 MeV), that contributes to the neutron dose outside the Mevatron treatment room; neutrons that crossed the 1 m concrete barrier had higher energies than those behind the 1.7 m concrete wall (with energy below 1 keV).

In order to compare the neutron detection parameters r_e_ and k_e_, we divided neutron energy E_c_ (MeV) of the spectrum (Figure 5) in the two areas as follows: first area E_c1_ < 2 MeV, and the second one 2 MeV ≤ E_c2_ ≤ 7.5 MeV.

Afterwards, we estimated the respective mean cross-sections σ_t_ = σ_c1_ = 0.619 b (for thermal neutrons, like above), σ_c2_ = 0.203 b (for energies E_c1_, E_c2_, respectively) from the curve σ(E) in Figure 1, and we determined the neutron detector responses for the three energy areas as follows: σ_c1_/σ_c2_ = r_c1_/r_c2_, where r_c1_ = r_t_ = 7.94×10^−4^ was the response that had already been calculated by using the equation [1]. Thus we got r_c2_ = 2.6 × 10^−4^ and we were able to determine the average or effective value of the neutron detector response r_ce_, but as a weighted or pondered mean.

Therefore, we took the surfaces under the neutron fluence spectrum curve (Figure 5; f(E)) for the two energy regions, like above, and we got the relative surfaces: s_c1_ = 0.22, s_c2_ = 0.78, which had the meaning of the relative frequencies in the calculation of the pondered mean. The mean or effective detector response was: r_ce_ = r_c1_s_c1_ + r_c2_ s_c2_ = (3.78 ± 0.03) ×10^−4^.

Having considered a great depending *k* on neutron energy (Figure 3), we calculated the average or effective *k*_ce_ for the three energy areas, and we used the same relative frequencies s_ci_ (i = 1, 2, 3); taking the average values of *k*_c_ for the neutron energies E_c1_ and E_c2_ as k_c1_ = 366, k_c2_ = 433 pSv cm^2^, respectively, we calculated the effective conversion factor as follows:

$${\text{k}}_{\text{ce}}={\text{k}}_{\text{c1}}{\text{s}}_{\text{c1}}+{\text{k}}_{\text{c2}}{\text{s}}_{\text{c2}}=418\;\text{pSv }{\text{cm}}^{2}.$$

Thus, since k_c_/r_c_ = 25,75 k_b_/r_b_, and according to equation [2], one can see that neutrons of the given distribution in Figure 5 (behind the 1 m concrete barrier) contribute to a neutron dose 26 times more than neutrons from the distribution in Figure 4 (behind the 1.7 m concrete wall).

## Conclusions

The neutron dosimeter, consisting of the LR-115 track etch detector and boron foil BN-1, was calibrated on thermal neutrons generated in the nuclear reactor of the J. Stefan Institute (Slovenia), and was applied to dosimetry of undesirable neutrons in photon radiotherapy with the Siemens Mevatron 15 MV electron linear accelerator.

Having considered a broad neutron spectrum of energies in the photon beam and high dependence of the track detector response on neutron energy, we divided the spectrum in the two energy areas, below and over 2 MeV. Afterwards we determined the detector responses (r) for the energy areas using corresponding cross-sections for neutron and boron, and then we calculated the pondered or effective response depending on surfaces under the respective neutron spectrum areas. Using the empirical curve k(E), we performed the similar procedure for determining an effective conversion coefficient (*k*) from neutron fluence to dose equivalent, depending on neutron energy (E).

The relative measurement errors made by track etching method with the LR-115 detector were about 1%. It is to mention that we took the data from the empirical curves (e.g. σ(E), k(E)) as average values without respective experimental errors.

The measurement of the neutron dose equivalent by the track etch detector, positioned outside the Siemens Mevatron 15 MV room (room wall of 1.7 m, density of 3200 kg/m^3^), gave the dose rate of 0.62 μSv/h.

Observing the neutron spectrum attenuation from 15 MV Mevatron behind the 1m concrete barrier, one was able to notice that the neutrons (Figure 5) had higher energies and contributed to a neutron dose 26 factor times more than neutrons from the distribution in Figure 4 (behind the 1.7 concrete wall).
